# The psychobiology of child and parental stress and the subjective perception of parental stress in a clinical sample of children

**DOI:** 10.3389/frcha.2023.1173317

**Published:** 2023-10-03

**Authors:** Annika Melinder, Astrid Brænden, Andrea Lebena, Åshild Olsen Faresjö, Elvar Theodorsson, Marit Coldevin, Jan Stubberud, Pål Zeiner

**Affiliations:** ^1^ Department of Psychology, University of Oslo, Oslo, Norway; ^2^ Division of Mental Health and Addiction, Oslo University Hospital, Oslo, Norway; ^3^ Department of Health, Medicine and Caring Sciences, Faculty of Medicine and Health Sciences, Linköping University, Linköping, Sweden; ^4^ Division of Clinical Chemistry and Pharmacology, Department of Biomedical and Clinical Sciences, Faculty of Medicine and Health Sciences, Linköping University, Linköping, Sweden; ^5^ Lovisenberg Diakonale Sykehus, Oslo, Norway; ^6^ Institute of Clinical Medicine, Faculty of Medicine, University of Oslo, Oslo, Norway; ^7^ Department of Research and Development, Clinic of Mental Health and Addiction, Oslo University Hospital, Nydalen, Norway

**Keywords:** PSI, hair cortisol, child, treatment strategies, multidiagnostic sample

## Abstract

Parental stress may influence the assimilation of treatment strategies and affect a child's recovery trajectory. Thus, assessing parental stress is crucial for children requiring psychiatric care. The Parenting Stress Index (PSI) is widely utilized to gauge perceived parental stress. However, since the PSI does not quantify cortisol concentration (i.e., a biological marker for stress), it is vital to ascertain the alignment between these indicators. Moreover, understanding the correlation in cortisol concentrations between parents and children in clinical contexts can refine assessment and diagnostic methodologies. In an outpatient sample [mean age (*M*_age_) = 9.68 years], we examined the correlation between hair cortisol concentration (HCC) in 60 pairs of parents and children, analyzed the relationship between PSI scores and parent HCC (*n* = 65), and used a regression model to probe the influence of child HCC and PSI scores on parent HCC (*n* = 63). The results showed a significant relationship between parent and child HCC (*p* < 0.001). The “Distraction and Hyperactivity” PSI subscale correlated significantly with parent HCC (*p *= 0.02). None of the PSI scores correlated with child HCC (*p* ≥ 0.07). The regression model, accounting for 44% of the variance, demonstrated that only child HCC significantly predicted parent HCC (*p *< 0.001), while the “Distraction and Hyperactivity” subscale did not.

## Introduction

Every parent experiences stress associated with the transition to parenthood and the challenges of providing adequate care (1). When a child requires psychiatric assessment and intervention, this introduces an additional layer of stress, which can have consequential implications for physiological measures, such as parental cortisol production (2). The magnitude of this parental stress can potentially influence the assimilation of treatment strategies and directly impact the child's recovery trajectory (3). This is underpinned by the notion that unregulated stress may result in poorly adjusted emotional regulation and less functional parental behavior (4). Evidence suggests a bi-directional relationship between cortisol production and human behavior and mood, observable from infancy. This bi-directionality can be attributed, on one side, to a caregiver's capacity to regulate the stress of the infant, which is dependent on the caregiver's own abilities to regulate stress, subsequently shaping the infant's future stress regulation (5, 6). On the other hand, this mutual environmental exposure further shares genetic alleles underlying cortisol production (7). The associations between child and parental cortisol secretion are thus well established, and some studies show this link to be stronger in parents with adequate parenting skills and children showing fewer symptoms of attention and hyperactivity difficulties (8). The opposite direction has not been evinced. Accordingly, it is clinically important to measure and evaluate stress in parents with children in need of diagnostic evaluation and potential treatment at a child psychiatric unit.

A common instrument to capture perceived parental stress is the Parenting Stress Index (PSI) (9–11). Since PSI measures parents’ perception of stress, one criterion validity of the PSI could be a biological marker of stress, such as hair cortisol concentration (HCC). Cortisol extracted from hair samples is regarded as a reliable measure of stress over time (12, 13). In the present paper, we focus on this methodological and clinically important association in a sample of parents and children with emotional regulation difficulties referred to child psychiatric units. Addressing the association between PSI and HCC is important since the PSI is a widely used instrument in the clinic.

Before describing our research questions, the influence of cortisol production on human behavior and mood will be presented. Second, we consider the ecobiodevelopmental frameworks for child adversity (14), which suggests that early-life stressors in the environment of the child are incorporated into the biological system whereafter it becomes a bi-directional process that flips stressors back into the parenting. Third, we present an instrument capturing the subjective forms of parental stress (i.e., the PSI).

### The influence of cortisol production on human behavior and mood

Stress represents an important factor in the pathophysiology of a range of somatic diseases (13, 15, 16) and psychological conditions (17, 18). Increased secretion of glucocorticoid cortisol results from the activation of the hypothalamic–pituitary–adrenal (HPA) axis caused by stress. An extensive body of literature highlights the associations and impacts of cortisol and HPA-axis activity on cognitive and emotional functioning. A recent review by James et al. (19) provides a comprehensive look into this subject.

Early-life stress, for instance, can lead to changes in neuroendocrine functions. These alterations may predispose individuals to depression when confronted with stress. This could result from the inability of the associated neural pathways, responsible for emotional, neuroendocrine, and autonomic regulation, to adapt effectively to challenging situations (20). In addition, altered HPA-axis reactivity may contribute to the formation of specific symptoms in prevalent child psychiatric conditions, such as in attention-deficit hyperactivity disorder (ADHD) (21). Childhood abuse can also alter the HPA-axis function and affect perceived anxiety, with the impact varying based on the severity and frequency of maltreatment (22). There is also evidence suggesting that HPA-axis alternations across the lifespan. For instance, the association between hair cortisol and psychological distress was larger among adults who had experienced adverse childhood experiences compared to those who had not (23). Even less severe neglect, such as parental discord, is found to affect children’s cortisol levels (24).

Both heightened and diminished HPA-axis activity correlate with a myriad of psychiatric conditions and other health outcomes (25). The dose, type, and duration of the adverse experiences likely affect differently (26). Encouragingly, positive interventions, such as psychosocial support, can potentially recalibrate the stress-response system. This suggests that the HPA dysregulation is not a permanent state but can be reversed (27). For instance, in children with a cluster of disorders defined by a persistent pattern of defiant or rule-breaking behavior, their cortisol response pattern during stress was related to treatment outcome (28).

### Ecobiodevelopmental frameworks and child adversity

For a comprehensive theoretical understanding of the causal pathways leading to health disparities and the impacts of early stress, we draw upon the ecobiodevelopmental frameworks proposed by Shonkoff and Garner (14). This model posits that early-life stressors in a child's environment are integrated into their developing brain and biological systems. The frameworks also suggest an intricate interplay among the child's behavioral, emotional, and biological functioning. Specifically, through processes of physiological adaptation or disruption, these functions, in conjunction with the early-life stressors (e.g., less sensitive parental monitoring), set the stage for altered biological functioning and long-term health consequences (29).

Because of the environmental influence on children's development and the following assumed blunted cortisol production, children's response to parents’ stress through everyday interactions is assumed to not only affect their biological adjustment (24, 30, 31), but to also affect parental care capabilities and perceived stress.

Parenting stress may be experienced when there is an apperceived discrepancy between parenthood demands and personal resources in several areas of life connected to parenting (32). Perceived parental stress might be related to dispositions within the parent (e.g., negative life events and health problems), to the social and economic situation of the parents, or it may be evoked by the child's difficulties and special needs (33). In addition, parental stress may be fluctuating (i.e., managing a defiant toddler) or it can be chronic (i.e., persistent over weeks or even months). Fluctuating stress may accumulate, rendering some parents more susceptible to ineffective coping strategies and chronic stress (34). The parents of children referred to psychiatric clinics are likely exposed to considerably greater stress levels and, in certain instances, to persistent chronic stress, potentially predisposing them to harsh parenting.

Crucially, parents who perceive themselves as lacking the capability to oversee childcare or provide safe parenting may experience heightened stress. This can further diminish their cognitive and emotional capacity to constructively engage with their struggling child (34). This bi-directional process can perpetuate stressors within parental functioning, creating a self-reinforcing cycle. In fact, even in a general population sample, there was a strong association between elevated cortisol levels in children and increased stress experienced by parents (35). Children in need of psychiatric care often exhibit symptoms such as emotional reactivity, aggressive behavior, limited emotional control, difficulties with cognitive flexibility and severe irritability. These symptoms can intensify the demands on parents, making their caregiving role more challenging and potentially compromising their ability to cope with stress (36, 37). Specifically, the PSI child domain provides items that link these emotional, behavioral, and cognitive factors (e.g., distractibility/hyperactivity, adaptability, demandingness) to parental stress expressions. Furthermore, irritability is among the most common reasons why families are referred to child mental health services (38, 39). Emotional reactivity has thus been identified as a moderator to the otherwise strong relationship between parent and child HCC (40). Strong emotional reactivity in children is likely to trigger stress in parents, which in turn can hinder their ability to provide good enough parenting. The PSI parent domain includes items that uncover these feelings of inadequacy, including perceived lack of competence, feelings of isolation, role restriction, and depression. In the present study, two subscales from the Behavior Rating Inventory of Executive Function-2 (BRIEF2) (41) are used to gauge emotional reactivity: the emotional control and cognitive flexibility subscales.

While parental stress represents an important factor in daily life interaction and can pose long-term risks to a child's mental health (42), resilience factors have been observed to influence cortisol concentrations. For instance, greater parental sensitivity and fewer symptoms of ADHD in children have been found to increase the correlation between parent and child HCC levels (8). This could suggest that synchrony between the parent and child may hold greater significance than purely genetic perspectives.

### Parent-reports of parental stress and biological measurements of cortisol

The PSI is a self-report questionnaire that taps into specific domains of stress-related issues in parenting (for a detailed description, see the Methods section). Interestingly, the PSI has been employed in studies comparing PSI to child salivary cortisol in ADHD (43) and to child salivary cortisol and executive functions (44), examining interventions for the parents of preschoolers with ADHD (45) and community-based implementation of a parent–child interaction therapy (46), and in studies of children with neurodevelopmental disorders (47). Studies utilizing the PSI have also explored the relationship between parents’ expectations of mental health treatment and the outcomes of their children's treatment, showing that perceived parental stress decreased when satisfaction with treatment was positive (48). Regarding other types of parenting, the level of perceived stress in foster parents before and after a mentalization-based intervention has been examined with the PSI (49). There is evidence of a strong relationship between perceived parental stress when measured with PSI and child atypical behavior or pathology: in samples of prenatal drug-exposed children (50); in children with autism spectrum disorder (51); in moderating the effect of neglect (52); and in children with ADHD (45), to mention a few of the studies using PSI.

While the norm data on PSI from the USA is extensive, there is a lack of norms in other cultures. The available Norwegian norm data are scarce but indicates that the population reports lower stress on all scales compared to the samples from the USA (53). Furthermore, the internal consistency and construct validity are regarded as good, but there is no criterion validity documented for the Norwegian version (54). One such criterion could be a biological marker of stress, such as hair cortisol. In fact, there are few, if any, reports in general, even from the samples from the USA, addressing the association between PSI and cortisol.

Cortisol extracted from hair samples is regarded to be a reliable measure of stress over time (12, 13). Studies show a positive association between child and parental HCC from infancy (5, 6), during the preschool years, and throughout childhood (55). The intergenerational transmission of parent and child HCC may partly be due to shared genetic alleles, as shown in a study by Tucker-Drob et al. (7), in which genetic factors accounted for about half the variation in HCC of long-term cortisol exposure. Children and their parents are, not surprisingly, also exposed to similar stressors in daily life that may contribute to comparable cortisol exposure (56). Likewise, a study found the association between child and parent HCC to be moderated by the child's emotional reactivity, which indicates that less emotionally reactive children are less susceptible to parental variation in sensitivity, when tested in a community-based sample (8, 40). This underscores the potential of individual differences affecting the bi-directional influence and the transactional perspective on development.

### The present study

Stress, as described above, is a bi-directional phenomenon. Dysregulated child behavior and stress may affect parental stress and cortisol production, which may conversely influence parenting quality. In addition, parental sensitivity can affect the synchronicity between child and parent. Thus, it is important to adopt assessment tools that can inform interventions aimed at reducing parental stress. The PSI is the most common measure of perceived parental stress. Yet, the PSI has not been evaluated in terms of biological markers in parental HCC and related to child HCC. Considering the widespread use of PSI as a presumed valid indicator of parental stress, there is a need to relate the measures of child and parent HCC as markers of stress with PSI reports. First, we therefore investigate the association between parent and child HCC in a clinical sample. Second, we assess the association between parental HCC (as a stress biomarker) and PSI scores (as perceived parental stress). Third, we explore the influence of child HCC and perceived parental stress (PSI) on parental HCC. Based on the literature reviewed, we anticipate a correlation between parental and child HCC. However, due to the limited research on the interplay between PSI and HCC in both children and parents, our approach to the latter two objectives is primarily exploratory.

## Methods

### Participants

**Figure 1:**
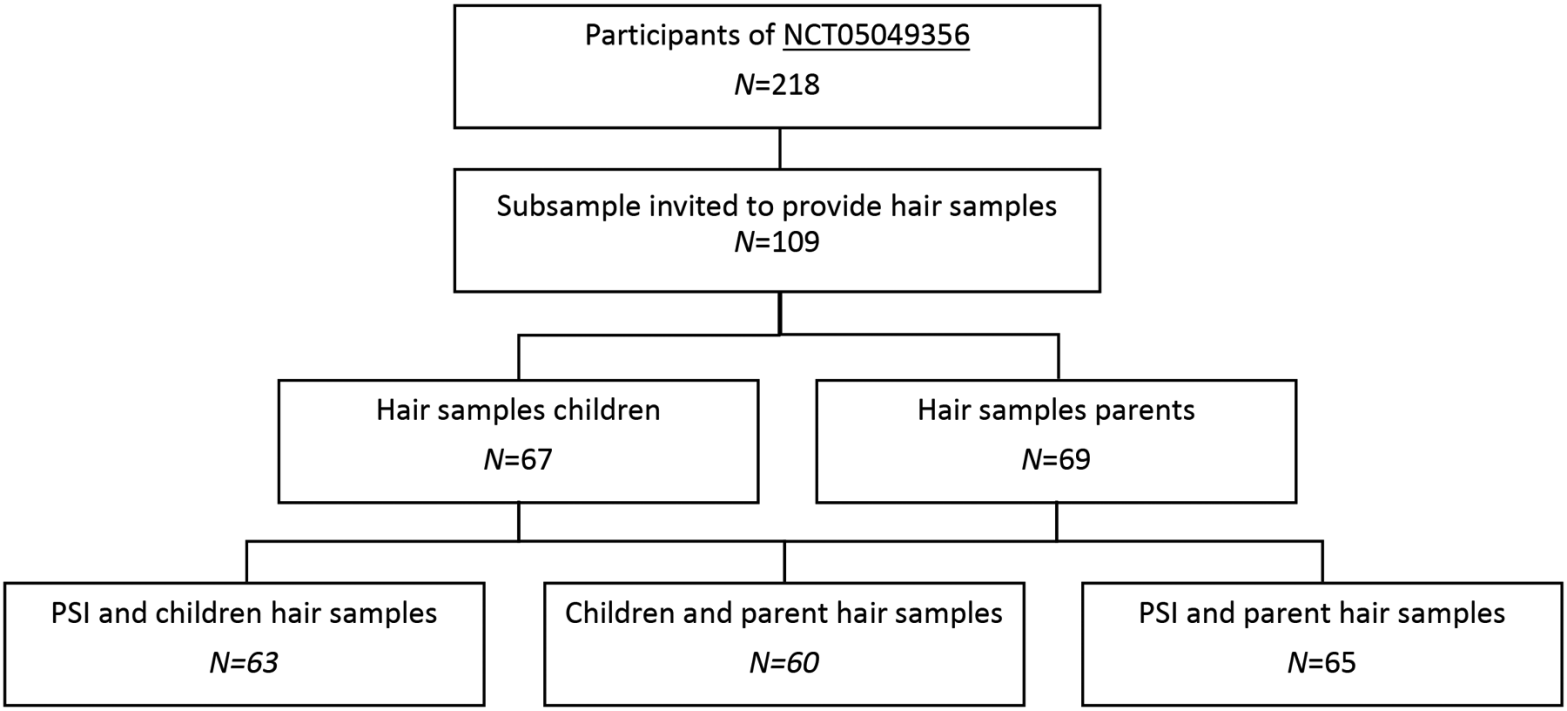
Flow-chart of participants recruitment and inclusion.

The participants were recruited among primary school attendants referred to the outpatient departments of child and mental health clinics in Oslo between January 2019 and August 2021 to take part in a larger study of underlying mechanisms in severe emotional dysregulation. Participants included a multidiagnostic treatment-seeking sample of 218 children, that is, these children did not receive psychiatric treatment or medication at the time of inclusion and study participation. The most frequent reasons for the referral were complex difficulties in the child's various life arenas (e.g., school, home, free time) and could consist of emotional regulation difficulties, cognitive challenges, and interpersonal conflicts and difficulties. For a description of clinical diagnoses, including comorbid conditions, see Brænden et al. (57). Informed and signed consent was attained from parents. Inclusion criteria included children aged 6–12 years, IQ ≥70, non-psychotic symptoms, and Norwegian language skills good enough to respond to a questionnaire and semi-structured clinical interview. Language skills in reading and responding to the questionnaires and a semi-structured clinical interview were the inclusion criteria for parents. A subsample (*n* = 109) from this study was additionally invited to provide hair samples for cortisol analyses (e.g., the child and the child's respective parents). Following this invitation, approximately 63% of the invited parents (*n* = 69) consented to participate. Two of the children could not provide hair samples, leaving 62% (*n* = 67) of the invited children for further analysis. The PSI was answered by the parents after informed consent was provided. In this process, 63 children and their parents provided hair samples *and* parental PSI, while in 60 cases we got HCC from *both* the child and the parent. Finally, parents’ HCC and their PSI responses were found in 65 cases. Because of that, analyses of our research questions include different participant numbers. See Figure 1 for a detailed depiction of the recruitment, inclusion of participants, and omitted data.

Both the main study and the present project were approved by the Regional Committee for Medical Research Ethics (2017/135) and are part of the registered study protocol (NCT05049356). We further employed data from a sample of typical developing children (e.g., they did not receive assessment or treatment from the healthcare section) (*n* = 87). This sample was recruited for a different study (58) by the same laboratory that performed the HCC analysis in the current clinical sample using the exact same radioimmunoassay (RIA) method (see below). This group was used as our reference group of non-clinical children regarding hair cortisol concentration only.

### Procedures

Participants were partly recruited between January 2019 and August 2021. Parents agreeing to participate in the study signed an informed consent form provided by the respective clinician responsible for the clinical assessment, on behalf of themselves and their child. To ensure children's rights to withstand from participating against their will, an orally age-adjusted version of the general information pamphlet was given to the child. In addition to the regular clinical examination, the assessments involved the children providing their hair samples and the parents filling out the PSI in addition to providing hair samples and demographic information. All data collection was done in one session. For the exact procedures, see the section below. Testing of those during the pandemic phase was adjusted in accordance with the hospitals’ recommendations and implied the use of masks and non-physical contact. However, all participants who provided hair samples were physically approached by the clinicians in charge. Hair samples were sent to the Department of Health, Medicine and Caring Science and Public Health, at Linköping University, Sweden, for analysis.

### Materials

#### Hair sampling and HCC analysis

Hair was cut from the vertex posterior of the scalp close to the skin by trained clinical psychologists in concurrence with the diagnostic evaluation, in accordance with the recommendations of the Society of Hair Testing (59), and because this area has a consistent growth rate (60–62). Each hair sample had a minimum length of 3.0 cm, representing exposure to stress approximately 12 weeks in the past. The method applied for the extraction and analysis of cortisol levels in hair was a competitive RIA, which enabled us to analyze small samples (63, 64). The hair samples were cut into smaller pieces, frozen for 2 min in liquid nitrogen, and minced together with a 5 mm steel ball using a Retch Tissue Lyser II for 2 min. Methanol (1 ml) was added to each tube and the samples were extracted overnight on a moving board. Then, 0.8 ml of the methanol supernatant was pipetted off and lyophilized using a Savant Speed Vac Plus SC210A. The lyophilized extracts of hair samples were dissolved in 300 μl 0.1 mol/L phosphate buffer, pH 7.4, containing 0.02% bovine serum albumin and 0.01% triton X-100, and cortisol concentrations were analyzed as described by Morelius et al. (65). Hair samples of 5 mg or more were needed to maintain a total inter-assay coefficient of variation below 8% for hair extraction and measurement of cortisol by the radioimmunoassay. The intra-assay coefficient of variation for the radioimmunoassay itself was 7% at 10 nmol/L (63). Furthermore, the gamma radiation used for detection in the RIA method is not influenced by colored hair, which is extracted to the methanol together with the cortisol. The research protocol and all methods in the study were carried out in accordance with relevant guidelines and regulations.

The PSI (9, 10), version 3, is translated into Norwegian and contains two domains (child and parent) that form a total scale of 101 items to which the parents reply on a Likert scale ranging from 1 (not agree at all) to 5 (totally agree). There is also a life stress scale of 19 items, which provides information about the parental stress experienced outside the relationship with the child. Six subscales (Distractibility/Hyperactivity, Adaptability, Reinforces Parent, Demandingness, Mood, and Acceptability) constitute the child domain, whereas seven subscales (Competence, Isolation, Attachment, Health, Role Restriction, Depression, and Spouse/Parenting Partner Relationship) are measure parent characteristics. Parents respond to the two domain scales as they experience their child (e.g., “My child is so active that I get exhausted”) and how they experience their own function (e.g., “Having got a child has changed my sleeping habits”), that is they respond to parental stress in relation to their child's behavior and in relation to how they feel in life and as a parent.

Test–retest reliability is in the range of 0.55–0.82 for the child domain, 0.69–0.91 for the parent domain, and 0.65–0.96 for the total stress score (66). Validity has been investigated in studies that focused on at-risk children, attachment, ADHD, child abuse, forensic contexts, medical treatment adherence, substance abuse, and parental depression, among others (66). The Norwegian version was translated by Kaaresen et al. (67). The available norm data are limited but indicate that the Norwegian female sample (*n* = 754) attains lower scores on all scales compared to the samples from the USA (53). Note that the Norwegian sample was extremely homogenous (e.g., only mothers of 12-month-old infants). No criterion validity is ever documented in the Norwegian version (54). In the present study (cases having hair samples from parent and PSI, *n* = 63), Cronbach's alpha for all subscales was 0.88 (total scales, *n* = 13), 0.82 for the child domain (*n* = 6), and 0.85 (*n* = 7) for the parent domain.

The BRIEF2 (41) is a standardized questionnaire consisting of 63 items measuring children's executive functions in daily life based on parent evaluation. Parents respond (1) never, (2) sometimes, or (3) often. Items are aggregated into three indexes reflecting Emotion Regulation (ERI), Behavior Regulation (BRI), and Cognitive Regulation (CRI), in addition to a total executive function composite [Global Executive Composite (GEC)]. To reflect emotional reactivity, we employ the ERI index, which is composed of two subscales: emotional control (e.g., has severe temper outbursts) and cognitive flexibility (e.g., has difficulty getting used to new situations). The raw score range of ERI is 16–48, and higher scores indicate worse functioning. For the present data, Cronbach's alpha demonstrated acceptable internal consistency on the ERI index = 0.8.

### Statistical analysis

All data were analyzed using SPSS Statistics version 26 (IBM Corp., Armonk, NY, USA). Demographic characteristics were analyzed using a one-way analysis of variance (ANOVA). HCC was logarithm-transformed before ANOVAs were conducted because the data were not normally distributed. To assess the role of child HCC on the association between parental HCC and their scores on the PSI measure, we first ran a set of bivariate correlations assessing the overall associations between the variables of interest. Next, we ran a regression analysis for parental HCC outcome—only including variables that were proven significantly correlated in the previous analyses. We also calculated quintiles of HCC (13, 16). Differences between children and parents concerning the distribution of quintiles were analyzed using the chi-square test. Two-tailed probabilities were used for testing statistical significance, which was defined as *α* = 0.05. For effect sizes, we use Cohen's *d*, which we interpret as 0.20 = small, 0.50 = medium, and 0.80 = large effects (68).

## Results

### Preliminary analyses

All demographics are shown in Table 1. The parental situation illustrates a relatively typical Norwegian family income (1 Euro is equivalent to approximately 10 NOK) where the child is living. Likewise, whether the child is living with one parent or with both parents together appeared to be similar to the typical Norwegian context. Slightly more boys participated in the study sample, whereas in the reference group of non-clinical children the sex distribution was more equal. Compared to the reference group of non-clinical children, the study sample's age was older [*F* (1,154) = 41.87, *p* < 0.001]. Note that HCC was logarithm (log) transformed for further analyses. As expected, the study sample and reference group of non-clinical children differed in terms of HCC [*F* (1,152) = 56.86, *p* < 0.001].

**Table 1:** Demographic information, HCC, and ERI.

	Clinical group	Non-clinical group
	*n *= 67	*n *= 87
Girls, *n* (%)	27 (40)	48 (55)
Age, *M* (SD)	9.68 (1.73)	6.41 (3.84)
Age, range	6.2–12.6	1.0–14.0
Total family income, *n* (%) (NOK)		
<700,000	8 (13)	—
>700,000	53 (87)	—
Living situation, *n* (%)		
Shared	51 (76)	—
Common	15 (22)	—
HCC (pg/mg), *median*		
Children total	87.05^a^	23.4
Girls	87.62	19.0
Boys	85.73	30.1
Parents	81.91^b^	—
HCC (log-transformed), *M* (SD)		
Children total	1.96 (0.47)	1.43 (0.40)
Girls	1.96 (0.51)	1.38 (0.42)
Boys	1.95 (0.45)	1.48 (0.37)
Parents	2.00 (0.45)	—
Emotion regulation, M (SD)		
EC girls	18.46 (4.16)	—
EC boys	19.32 (4.41)	—
CF girls	15.12 (4.21)	—
CF boys	16.46 (4.13)	—
ERI girls	33.58 (7.95)	—
3ERI boys	35.68 (7.88)	—

EC, emotion control; CF, cognitive flexibility.

Living situation: common = the child lives full-time with both parents. Shared = the child lives part-time with each parent.

^a^ HCC non logarithm transformed cortisol levels children (Q1 very low; 9.78–32.90, Q2 low; 33.27–57.40, Q3 moderate; 59.77–114.63, Q4 high; 125.18–225.07, Q5 very high; 241.40–1,610.10).

^b^ HCC non logarithm transformed cortisol levels parents (Q1 very low; 20.04–40.66, Q2 low; 44.22–64.85, Q3 moderate; 66.30–119.37, Q4 high; 124.53–198.35, Q5 very high; 228.07–3,193.33).

Preliminary analysis revealed no significant main effects involving the children’s sex on child HCC [*F* (1,65) = 0.01, *p* = 0.93], on the ERI measures [*F* (1,65) < 0.63, *p* ≥ 0.20], or on parental HCC [*F* (1,59) = 1.13, *p* = 0.29]. Except for the subscale “Accept” [*F* (1,63) = 7.87, *p *< 0.01 (*M*boy = 15.30, *Sd*boy = 3.75, *M*girl = 12.63, *Sd*girl = 3.60)], the children’s sex did not significantly influence any of the remaining PSI subscales or total PSI scale measure [*F* (1,63) ≤ 0.3.01, *p* ≥ 0.09]. Data were therefore collapsed across child sex in further analyses of HCC, ERI, and PSI, except for the scale “Accept.” The children's age was significantly negatively correlated with their HCC (*r* = −0.45, *p* < 0.001, *n* = 67), indicating a decrease in HCC with older age. We did not detect any age-significant association in the reference group of non-clinical children (*r* = −0.14, *p* = 0.19, *n* = 87). However, note that the same direction of the correlation was found.

### Main analyses

**Figure 2:**
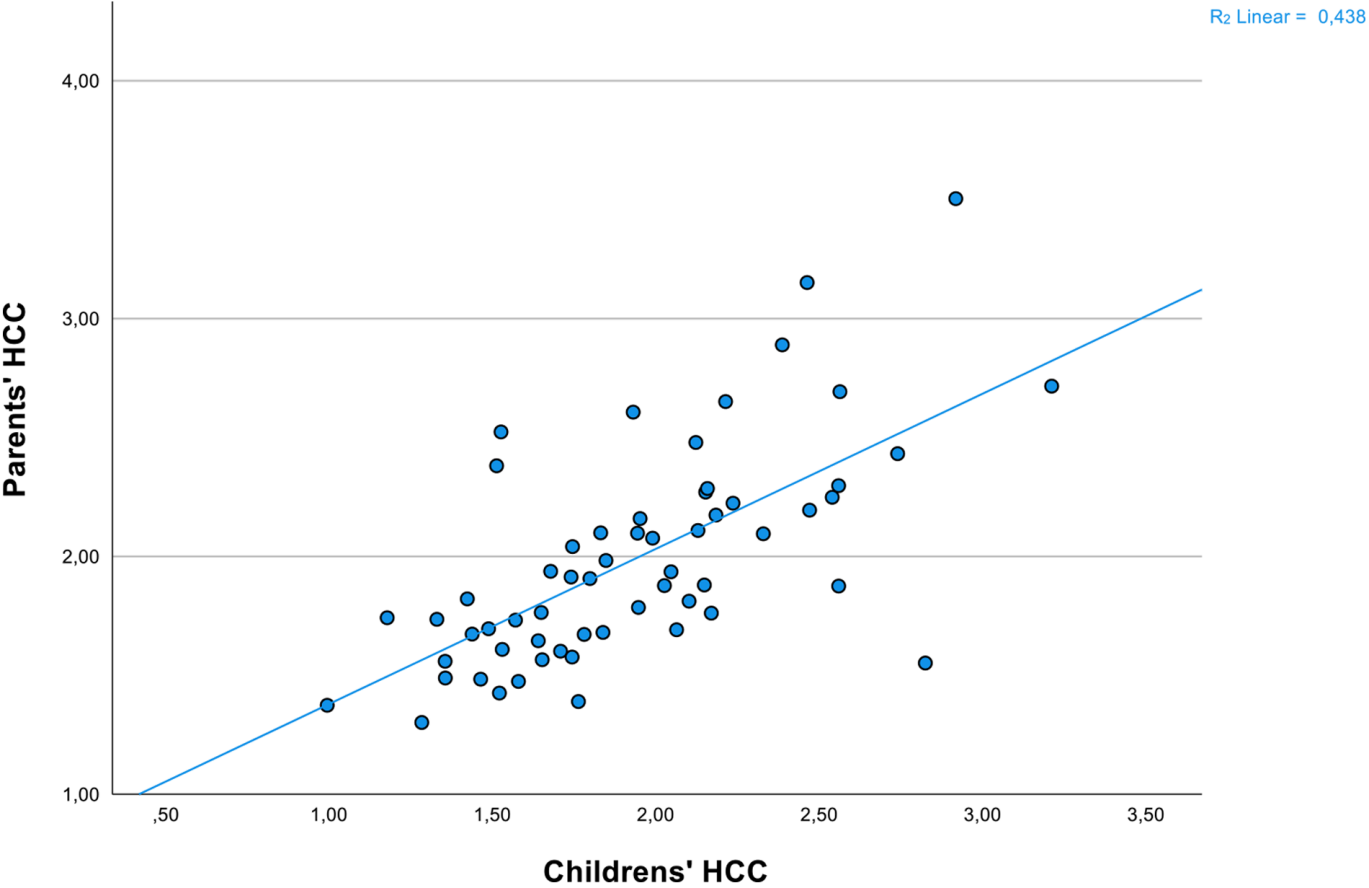
Scatterplot of log-transformed HCC from children and parents. Scatterplot of natural logarithm-transformed HCC from the clinical children (*n *= 60) and their parents (*n *= 60).

Confirming our first hypothesis, child and parent HCC levels were significantly associated (*r* = 0.66, *p* < 0.001, *n* = 60) (Figure 2). As this relationship has been found to be moderated by the child's emotional reactivation, we employed the two subscales of the ERI index (i.e., emotional control and cognitive flexibility) from BRIEF2 as predictor variables in a linear regression with child HCC as the dependent factor and parent HCC as the independent factor. The model was significant [*F* (3,56) = 16.58, *p *< 0.001, *R*^2^ = 0.47]. While emotional control did not contribute to the outcome of child HCC [*β* = −0.23, *t*(59) = −1.50, *p *= 0.14], a non-significant tendency appeared for cognitive flexibility [*β* = 0.28, *t*(59) = 1.84, *p *= 0.07]. Parent HCC was thus the only significant predictor to child HCC in the model [*β* = −0.61, *t*(59) = 5.954, *p *< 0.001].

To explore the associations between parental HCC response and their reports of experiences of stress on PSI, we correlated all PSI measures with parental HCC. As can be seen in Table 2, only one of the PSI measures, “Distraction and Hyperactivity,” correlated significantly with the parental HCC measure (*r* = 0.29, *p *= 0.02, *n* = 65). The remaining subscales were not significant (*r* ≤ 0.13, *p* ≥ 0.32, *n* = 65). From Table 2, it is further read that child HCC and parental-rated stress PSI variables did not correlate (*r* ≤ 0.23, *p* ≥ 0.07, *n* = 63).

**Table 2:** Correlations between PSI scales and log-transformed HCC for children (*n* = 63) and their parents (*n* = 65) respectively.

		HCC (log)	HCC (log)
		Children	Parent
PSI distraction and hyperactivity	*r*	0.229	0.292
	*p*	0.072	**0** **.** **018**
PSI adjustment	*r*	0.066	0.056
	*p*	0.605	0.656
PSI reward	*r*	−0.140	−0.093
	*p*	0.274	0.463
PSI demanding	*r*	0.196	0.126
	*p*	0.123	0.317
PSI mood	*r*	−0.092	−0.067
	*p*	0.473	0.594
PSI accept	*r*	−0.036	−0.018
	*p*	0.777	0.886
PSI competence	*r*	0.036	−0.078
	*p*	0.778	0.537
PSI isolation	*r*	0.007	−0.076
	*p*	0.954	0.547
PSI attachment	*r*	0.053	−0.055
	*p*	0.680	0.664
PSI health	*r*	0.076	0.020
	p	0.552	0.875
PSI limited	*r*	0.094	−0.001
	*p*	0.463	0.994
PSI depression	*r*	0.068	0.045
	*p*	0.599	0.724
PSI spouse	*r*	−0.002	0.044
	*p*	0.987	0.726
PSI child domain	*r*	0.089	0.102
	*p*	0.489	0.418
PSI parent domain	*r*	0.104	0.014
	*p*	0.416	0.914
PSI total stress	*r*	0.111	0.066
	*p*	0.388	0.602
PSI life stress	*r*	−0.043	0.057
	*p*	0.735	0.654

Pearson coefficient is reported (*r*). *P*-value significant at ≤0.05. Number varies compared to the sample presented in Table 1 due to a reduction when integrate with PSI data as explained in Methods section.

Bold value indicates a significant *P*-value.

**Figure 3:**
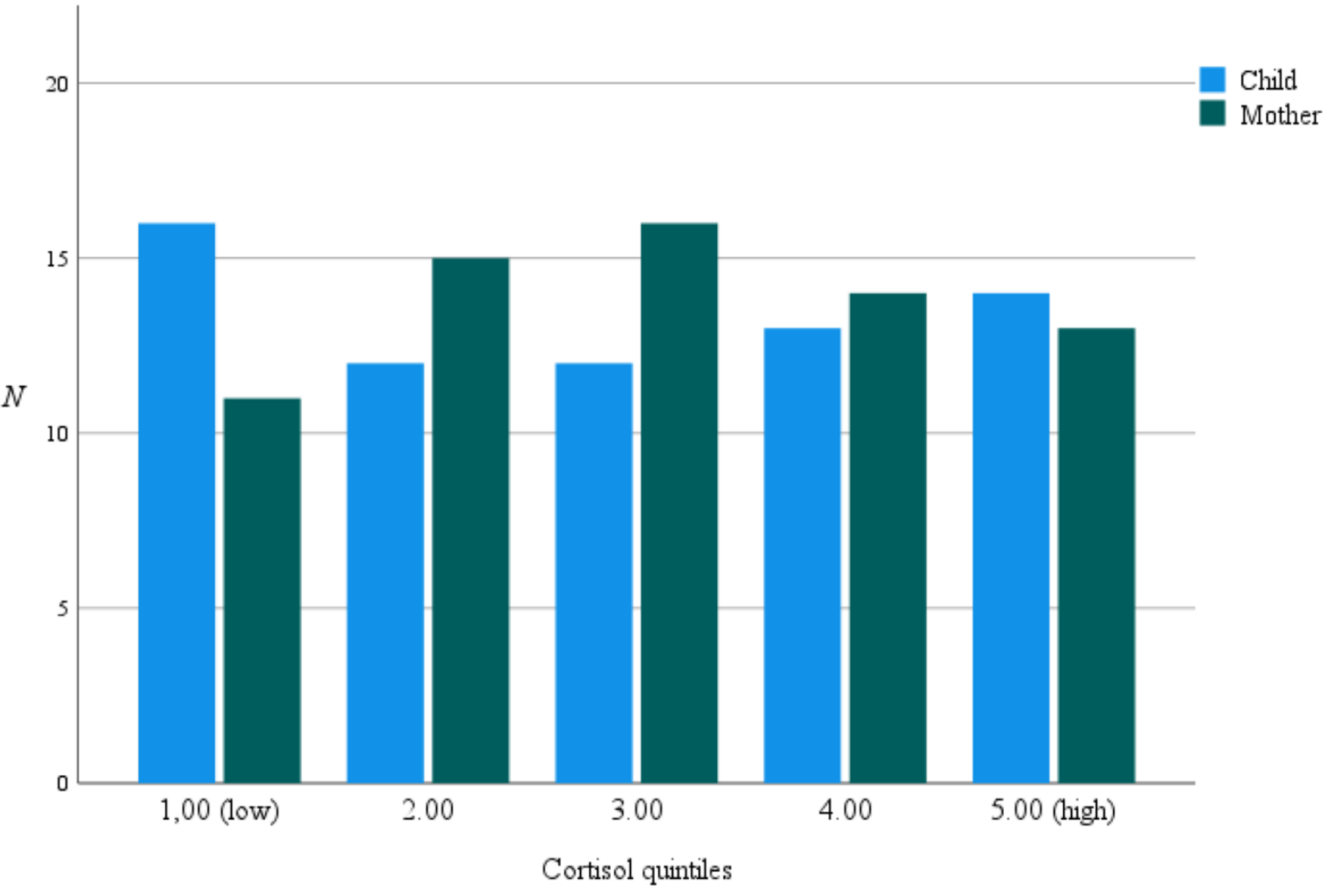
HCC distribution in quintiles. Distribution of HCC (pg/mg) in quintiles for children and parents collapsed: low (Q1): 9.8–36.8, median = 28, (Q2): 36.8–60.8, median = 48.1, (Q3): 60.9–124.7, median = 85.3, (Q4): 124.8–235.5, median = 149.3, (Q5): 235.6–3,193.3, median = 362.2.

The potential effects of children's HCC and the parents’ scores on experienced stress from PSI on parental HCC were lastly tested using a regression analysis with child HCC and the PSI's “Distraction and Hyperactivity” scale as predictor variables. The model was significant [*F* (2,53) = 20.74, *p *< 0.001, and *R*^2^ = 0.44]. The results revealed that child HCC significantly predicted scores on the parental HCC [*β* = 0.63, *t*(63) = 5.94, *p *< 0.001]. On the contrary, the PSI's “Distraction and Hyperactivity” scale was no longer a significant association, and did not significantly predict scores on parental HCC [*β* = 0.006, *t*(65) = 0.85, *p *= 0.40]. No differences in the distribution of HCC quintiles between children and mothers were detected [χ^2^ (4,136) = 1.88, *p *= 0.76]; for illustrative purposes, see Figure 3.

## Discussion

In the present study, we explored the association between parental and child HCC, hypothesizing that they would be related. Second, we examined the relationship between parental HCC and perceived parental stress (PSI). We also explored the potential effects of children's HCC and PSI on parental HCC. While our first hypothesis was validated, we found no significant correlation between parental stress and its biological markers.

Ecobiodevelopmental frameworks suggest an interplay between a child's behavioral, emotional, and biological functions (14). Despite the growing evidence associating early-life stressors with child hair cortisol, this potential interplay with child emotional functioning remains under-investigated. In line with the findings of the present study using a clinical sample, a previous study identified a positive association between child and parental HCC in a community-based group of children (40). Notably, Kao et al. found that child and parental HCC was moderated by the child's emotional reactivity. In the present study, the children's emotionality did not significantly impact our findings. However, cognitive flexibility emerged as a significant predictor. Comparing the instrument used by Kao et al. (40) with ours to measure emotionality (e.g., emotional reactivity), a high level of overlap evinced in terms of construct. For instance, both covered elements such as frustrations, mood swings, and displaying adequate negative affect when provoked. Given that the sample of the present study was clinical, contrasting the community-based group in the study by Kao et al. (40), it raises the possibility that biological predispositions might be more influential in psychological disorders, whereas psychological and family dynamics may be more pivotal in conventional/non-clinical cases. Heritability might significantly shape the origin of stress, especially in samples displaying psychological deviations (69). From this vantage point, the lack of association between PSI scales and child HCC in the present findings might suggest a reduced influence of psychological factors on child stress manifestations and vice versa. However, Schloß et al. (8) highlighted that environmental factors, such as maternal sensitivity, can influence the stress functioning in subgroups of children with symptoms of ADHD.

This does not mean that psychological intervention may not be efficient in reducing child symptoms and cortisol levels. One should, however, be attentive to the mismatch between parental-rated evaluations of a child's emotional, behavioral, and cognitive performance as a composite measure of parental stress. As HCC might serve as an indicator of psychological disorders in children (57), we suggest that clinicians should be attentive to this manifestation. Though current clinical evaluations do not typically measure HCC, incorporating it in the future could reveal abnormal cortisol levels. Such insights could highlight the complex interactions between biological and psychological processes and enable more tailored treatment plans, which could thereby potentially alleviate feelings of parental uncertainty and feelings of helplessness that perpetuate perceived stress.

Second, only the “Distraction and Hyperactivity” measure from PSI correlated with parental cortisol levels. While the PSI captures stress specific to parenting, HCC measures biological stress, presumably reflecting broader stressors beyond just parenthood. This knowledge could be useful during treatment as clinicians can assure parents that even if they experience ongoing stress with their children, it does not necessarily translate biologically, or at least alone not significantly change, cortisol levels. Of note, the level of cortisol in hair seems to relate to HPA-axis functioning over longer periods compared to saliva cortisol, which is why the HCC marker may be more suitable for quantifying relatively long-term (e.g., 24 months) stressors (70, 71). Perceived parental stress may align closely with specific stages of child development, indicating prolonged stress accumulation. The scale “Distraction and Hyperactivity,” however, signalizes difficulties that could easily disturb social interactions involving close relationships, such as those between children and their parents. Thus, a child's difficulties with distraction and hyperactivity may influence a parent's reactivity and underlying stress response, including cortisol levels, over time. This is somewhat like the findings of Cosan et al. (72), who found that children's teacher-reported symptoms of ADHD were associated with parents’ HCC, although parent-reported symptoms of ADHD and parents’ HCC were not.

Third, proving the importance of testing more than single predictors in a model, the association between “Distraction and Hyperactivity” (e.g., the only significant scale from the PSI on parental cortisol) and parental cortisol concentration ceased when child cortisol concentration was added into the regression. In terms of using hair cortisol concentration as a validation of subjective perception of parental stress, we must conclude that the PSI did not appear to be equivalent to parental biological stress in the present sample. However, the study has limitations that restrict firm conclusions.

Although we did not formulate any research questions about the relationship between our clinical children and the reference group of non-clinical children, the last observation was that the clinical group of children showed higher levels of HCC. A potential biological mechanism is that long-term stress exposure and trauma could lead to a blunted cortisol response (73), with a possible suppression of the HPA-axis activity. The opposite trend was found in our study. It should be said that the clinical group was older than the non-clinical group, which may be one contributing factor to this result. However, there are too few participants to draw any correct conclusions, and replications are needed to fully conclude our results regarding the relationship between PSI and HCC.

### Limitations

Although we initially recruited a high number of participants, only half of the sample was invited to provide biological material for cortisol analyses. Of those who did not get the invitation, a vast majority was referred during the COVID-19 pandemic (i.e., during spring 2020). Of those who got the invitation, only 37% did not consent. However, as we tested during the COVID-19 pandemic, there is a risk that the period itself has affected cortisol levels (74). The pandemic presented multifaceted challenges, ranging from health concerns to socioeconomic disruptions, all of which could have influenced stress levels across individuals, irrespective of any clinical conditions. While our study ensured uniformity by collecting all samples during this period, this context might have introduced an external factor that impacted the HCC levels of both parents and children. A potential avenue for future research could involve comparing these relationships across different time frames. We are aware of the PSI-4 version. However, as this most novel version was not translated into Norwegian, we employed the PSI-3 version, which is frequently used by Norwegian clinicians. Furthermore, we regrettably did not employ a measure of child emotion reactivity specifically. This restricted our possibilities to replicate and extend a former study [e.g., Kao et al. (40)] in which emotional reactivity was found to be a solid moderator of the association between child and parental cortisol concentrations. However, the emotional regulation index from BRIEF2 is regarded to be a valid instrument measuring emotion regulation that may be regarded as an equivalent to the specific measure of emotion reactivity employed by Kao et al. (40). Lastly, since we have a perspective on processes that plays out over time (e.g., bi-directional influences of biological factors and parenting), it would have been favorable to employ a longitudinal design testing HCC across time. As HCC samples might be perceived as intrusive, ethical considerations received a stronger weight in our judgment of design.

## Conclusion

Expanding former research, we demonstrated a relationship between parental and child hair cortisol concentrations in a clinical sample of children. The association was also valid when perceived parental stress was entered as a predictor to parental cortisol concentration. The self-reported stress level and biological measures of stress did not correlate.

## Data Availability

The datasets presented in this article are not readily available because the dataset is confidential until all analyses have been conducted. Requests to access the datasets should be directed to a.m.d.melinder@psykologi.uio.no.
